# Loss of Class III Phosphoinositide 3-Kinase Vps34 Results in Cone Degeneration

**DOI:** 10.3390/biology9110384

**Published:** 2020-11-07

**Authors:** Ammaji Rajala, Feng He, Robert E. Anderson, Theodore G. Wensel, Raju V. S. Rajala

**Affiliations:** 1 Department of Ophthalmology, University of Oklahoma Health Sciences Center, Oklahoma City, OK 73104, USA; ammaji-rajala@ouhsc.edu (A.R.); robert-anderson@ouhsc.edu (R.E.A.); 2 Dean McGee Eye Institute, Oklahoma City, OK 73104, USA; 3 Verna and Marrs McLean Department of Biochemistry and Molecular Biology, Baylor College of Medicine, Houston, TX 77030, USA; fhe@bcm.edu (F.H.); twensel@bcm.edu (T.G.W.); 4 Department of Cell Biology, University of Oklahoma Health Sciences Center, Oklahoma City, OK 73104, USA; 5 Department of Physiology, University of Oklahoma Health Sciences Center, Oklahoma City, OK 73104, USA

**Keywords:** phosphatidylinositol 3-phosphate, class III PI3K, Vps34, phosphoinositides, retinal degeneration, phosphorylation, cone photoreceptor cells

## Abstract

**Simple Summary:**

Cone photoreceptors are the class of neurons in the retina that support daylight and color vision. In humans and rodents, the cone photoreceptors constitute a small percentage of total retinal photoreceptors; in some retinal diseases, these cells malfunction over time and cease to work, and eventually die. Class III phosphoinositide 3-kinase, also known as vacuole protein sorting 34 (Vps34), generates phosphoinositide 3-phosphate (PI(3)P), a lipid molecule that transmits information inside of the cell. PI(3)P plays an essential role in removing injured cells, a process called autophagy, which maintains a healthy environment, as well as in protein trafficking inside of the cell. Furthermore, PI(3)P can act as a bridging molecule for proteins to bind to each other. We eliminated the class III phosphoinositide 3-kinase in mouse cones, which resulted in the loss of visual function and death of cone cells. Our studies suggest that PI(3)P generated by class III phosphoinositide 3-kinase is essential for cone photoreceptor function and survival.

**Abstract:**

The major pathway for the production of the low-abundance membrane lipid phosphatidylinositol 3-phosphate (PI(3)P) synthesis is catalyzed by class III phosphoinositide 3-kinase (PI3K) Vps34. The absence of Vps34 was previously found to disrupt autophagy and other membrane-trafficking pathways in some sensory neurons, but the roles of phosphatidylinositol 3-phosphate and Vps34 in cone photoreceptor cells have not previously been explored. We found that the deletion of Vps34 in neighboring rods in mouse retina did not disrupt cone function up to 8 weeks after birth, despite diminished rod function. Immunoblotting and lipid analysis of cones isolated from the cone-dominant retinas of the neural retina leucine zipper gene knockout mice revealed that both PI(3)P and Vps34 protein are present in mouse cones. To determine whether Vps34 and PI(3)P are important for cone function, we conditionally deleted Vps34 in cone photoreceptor cells of the mouse retina. Overall retinal morphology and rod function appeared to be unaffected. However, the loss of Vps34 in cones resulted in the loss of structure and function. There was a substantial reduction throughout the retina in the number of cones staining for M-opsin, S-opsin, cone arrestin, and peanut agglutinin, revealing degeneration of cones. These studies indicate that class III PI3K, and presumably PI(3)P, play essential roles in cone photoreceptor cell function and survival.

## 1. Introduction

Phosphatidylinositol (PI) is a component of phospholipids in the cell membrane and contains a D-myoinositol head group, a glycerol backbone, and two fatty acids at the C1 and C2 acyl positions of glycerol [1,2,3]. Phosphorylation of multiple free hydroxyls at the 3, 4, and 5 positions on the inositol ring of PI generates phosphorylated phosphatidylinositol phosphates, collectively called phosphoinositides (PIPs) [2,4,5,6]. The action of phosphoinositide kinases and phosphoinositide phosphatases, which can rapidly convert one specific PIP into another, results in the generation of seven distinct PIPs [7]. These seven PIPs serve as site-specific signals on membranes that recruit and regulate protein complexes at the interface of the cytosol [2]. The PIP signals are used for various functions, including signal transduction, cytoskeletal assembly, membrane binding, fusion, and cell survival [1,2,3,8].

Phosphoinositide 3-kinases (PI3K) are a group of enzymes that specifically phosphorylate PI at position 3 to generate 3′ or D-3 phosphoinositides [6]. These PI3Ks have been grouped into three distinct classes, depending on subunit interactions and substrate specificity: class I, class II, and class III PI3Ks [6]. Class III PI3K (also called vacuole protein sorting 34 (Vps34)) selectively phosphorylates PI to PI(3)P, but does not phosphorylate other PIPs [6]. The PI(3)P lipid generated by Vps34 plays an important role in endocytic membrane trafficking, canonical autophagy, and cell survival [9]. We previously demonstrated that class I PI3K is essential for cone photoreceptor survival and that ablation of either subunit of PI3K (regulatory p85α or catalytic p110α subunits) resulted in age-related cone degeneration [10,11]. Surprisingly, the ablation of class I PI3K in rod photoreceptors did not affect rod structure and function [5].

Conditional deletion of Vps34 in rods resulted in a failure in the fusion of endosomal and autophagy-related membranes with lysosomes that prompted the buildup of anomalous membrane structures and exhibited progressive loss of rods by 12 weeks [12]. Initially, these mice have normal structure and function of rod photoreceptors and normal trafficking of rhodopsin to the outer segments. However, these mice experience progressive rod degeneration by 12 weeks of age [12]. Vps34 has recently been shown to be essential for on-bipolar cell survival, and loss of this enzyme in these cells results in a significant loss of structure and function [13]. This study further highlighted that PI(3)P is necessary for the fusion of autophagosomes with lysosomes and maturation of late endosomes, as well as the fact that PI(3)P is needed for the maintenance of on-bipolar cell health [13]. Currently, there are no studies available on the role of Vps34 in cone photoreceptors. Thus, we conditionally deleted Vps34 in cones and examined the effect on the structure and function of these cells.

## 2. Materials and Methods

### 2.1. Antibodies

Polyclonal Vps34 antibody was obtained from Cell Signaling (Danvers, MA, USA). Polyclonal antisera to the p85α regulatory subunit of class I PI3K were obtained from Upstate Biotechnology, Inc. (Lake Placid, NY, USA). The goat secondary antibody was procured from Santa Cruz Biotechnology (Santa Cruz, CA, USA). Rabbit polyclonal anti-red/green cone opsin (M-opsin), anti-cone arrestin, anti-S-opsin, and rabbit and mouse secondary antibodies were obtained from Millipore (Billerica, MA, USA). Mouse monoclonal anti-Cre antibody suitable for immunohistochemistry was purchased from Abcam (Cambridge, MA, USA). Monoclonal 1D4 rhodopsin antibody was a kind gift from Dr. James F. McGinnis (University of Oklahoma Health Sciences Center, Oklahoma City, OK, USA). Peanut agglutinin (PNA) and secondary antibodies were purchased from Vector Laboratories (Burlingame, CA, USA). 4′,6-diamidino-2-phenylindole (DAPI) used for nuclear staining was procured from Invitrogen Molecular Probes (Carlsbad, CA, USA). The monoclonal anti-arrestin antibody was a kind gift from Dr. Paul Hargrave (University of Florida, Gainesville, FL, USA). The monoclonal glutamine synthetase (GS) antibody was purchased from Abcam (Cambridge, MA, USA). Polyclonal glial fibrillary acidic protein (GFAP) was purchased from Dako (Carpinteria, CA, USA).

### 2.2. Animals

Our study followed the National Institutes of Health (NIH) Guide for the Care and Use of Laboratory Animals and the ARVO Statement for the Use of Animals in Ophthalmic and Vision Research. The Institutional Animal Care and Use Committee (IACUC) at the University of Oklahoma Health Sciences Center approved all protocols (Protocol # 18-033-CHITW). We are grateful to Dr. Anand Swaroop (NIH, Bethesda, MD) for providing the neural retina leucine zipper gene (Nrl^−/−^) breeding pairs, which produced the experimental animals used in this study. Mice born in our vivarium were raised in dim cyclic light (40–60 lux, 12 h light/dark cycle). Vps34-floxed mice [14], which have lox P sites flanking exons 17 and 18 (the ATP binding domain), were a kind gift from Dr. Fan Wang (Duke University, Durham, NC, USA)). We screened Vps34-floxed and CC-Vps34 knockout (KO) mice for rd1 and rd8 mutations. All mice were negative for these mutations. The generation and efficiency of rhodopsin-Cre [12,15] and human red/green pigment cone-Cre [11,16] mice have been described previously. The eyes or retinas were harvested after CO_2_ asphyxiation. These tissues were subjected to biochemistry or immunohistochemistry. Ground squirrel retinas were provided by Dana Vaughan (University of Wisconsin, Oshkosh, Oshkosh, WI, USA).

### 2.3. Preparation of Cone Photoreceptor Cells by Density Step-Gradient Centrifugation

Cone photoreceptor cells were isolated by a method we previously described [17]. Briefly, 28 cone-dominant *Nrl^−/−^* retinas were placed in ice-cold Ringer’s solution [10 mM 2-[4-(2-hydroxyethyl)piperazin-1-yl]ethanesulfonic acid ( HEPES) (pH 7.4), 130 mM NaCl, 3.6 mM KCl, 12 mM MgCl_2_, 1.2 mM CaCl_2_, and 0.02 mM ethylenediaminetetraacetic acid (EDTA)] containing 8% OptiPrep and were gently vortexed for 1 min. We repeated this process 5 times. The pooled crude lysate was placed on top of a 10, 15, 20, 25, and 40% OptiPrep step gradient. After centrifugation (19,210× *g* at 4 °C for 60 min), we collected 20 fractions from top to bottom, which were examined by immunoblots. We repeated these experiments 3 times. Each time, we observed consistent results in terms of fractionation.

### 2.4. Determination of PI(3)P Levels in Cone-Dominant Nrl^−/−^ and Ground Squirrel Retina

The phosphoinositides were extracted according to the method described earlier [12,18]. Retinas were homogenized in phosphate-buffered saline (PBS) and the lipids were extracted twice with chloroform/methanol (1:2) to remove the bulk of the phospholipid, and both fractions were pooled into a glass tube. This fraction corresponds to phospholipids (PL). To the remaining mixture, chloroform/methanol/H_2_0 (2:4:0.1) was added to extract the phosphoinositides. We repeated this process twice by adding chloroform and HCl, and all of the chloroform layers were pooled. The samples were then extracted with 1 mL of chloroform/methanol/12N HCl (2:4:0.8, v/v/v), vortexed, and centrifuged as above, and the lower chloroform phase was transferred to a glass tube. The chloroform/methanol/HCl extraction was repeated twice. This fraction corresponds to phosphoinositides (PI). The PL and PI pooled fractions were dried under nitrogen gas and the lipids were dissolved in chloroform/methanol (1:9). Lipid phosphorous content was measured using an inorganic phosphorous assay as described [19], and the lipid phosphorous was converted to a phospholipid amount [20]. The PI(3)P levels were measured using an ELISA assay [12] by coating various concentrations of PI(3)P in phosphatidylcholine (PC)/phosphatidylethanolamine (PE)/phosphatidylserine (PS) (50:35:15) on a 96-well plate (Immulon 2 HB) with PL and PI samples extracted from *Nrl^−/−^* and ground squirrel retina. Plates were air-dried under a hood at room temperature. Wells were then blocked with 3% bovine serum albumin (BSA) in PBS before incubation overnight with a purified PI(3)P binding protein, the GST-2X-Hrs-1D4 fusion protein. Wells were washed with wash buffer (PBS containing 0.05% Tween-20) and then incubated with a mouse monoclonal rhodopsin 1D4 antibody for 2 hours at room temperature. The plate was washed with wash buffer and the wells were incubated with anti-mouse horseradish peroxidase (HRP)-conjugate for 60 min at room temperature. Luminescence was detected using an ELISA plate reader. A standard curve was generated, and x values for the unknowns were generated from the slope equation. The concentration of phosphoinositide in PL and PI fraction was normalized to phospholipid and expressed as fmol/nmol phospholipid.

### 2.5. Generation of Cone-Vps34 Knockout Mice

To determine the functional role of Vps34 in cones, we mated exon 17 and 18 floxed Vps34 mice with mice expressing Cre-recombinase under the control of human red/green pigment (L/M opsin gene) promoter [11]. The resultant offspring were heterozygous for Vps34, with and without Cre expression. The heterozygous Vps34 mice carrying Cre were backcrossed with homozygous floxed Vps34 mice, which yielded the final genotypes of cone Cre Vps34 knockout (CC-Vps34 KO), with floxed Vps34 littermates as controls. The genotype of the CC-Vps34 KO mice (i.e., animals carrying the *cre* transgene and homozygous for the Vps34-floxed allele) was confirmed by PCR analysis of tail DNA. For detection of Cre, we used sense: 5′ – GCC GCA TAA CCA GTG AAA CAG CAT -3′ and antisense: 5′ – TTG GTT CCC AGC AAA TCC CTC TGA -3′ primers to generate a product size of 500 bp. To distinguish the Vps34-floxed allele from wild-type, we used three primers: A1: 5′-GGCCACCTAAGTGAGTTGTG-3′, A2: 5′-GAAGCCTGGAACGAGAAGAG-3′, and A3: 5′-ATTCTGCTCTTCCAGCCACTG-3′ primers to generate a 580 bp wild-type allele, a 430 bp floxed allele in heterozygous mice, and a 430 bp homozygous floxed allele, respectively. We screened floxed Vps34 control and CC-Vps34 KO mice for rd1 and rd8 mutations. All mice were negative for these mutations.

### 2.6. Immunohistochemistry and Immunoblot Analyses of Retinas and Cone Photoreceptor Membranes

Immunohistochemistry and immunoblot analysis were performed as previously described [21]. In the current study, blots were incubated with anti-M-opsin (1:1000), anti-p85α (1:1000), anti-Vps34 (1:1000), anti-rhodopsin (1:10,000), anti-rod arrestin (1:1000), anti-S-opsin (1:1000), anti-cone arrestin (1:1000), and anti-actin (1:1000) antibodies overnight at 4 °C. The blots were then washed and incubated with HRP-coupled anti-mouse or anti-rabbit secondary antibodies (as appropriate) for 60 min at room temperature. After washing, blots were developed with enhanced SuperSignal West Dura Extended Duration Substrate (Thermo Fisher Scientific, Waltham, MA, USA) and visualized using a Kodak Imager with chemiluminescence capability. The original blots can be found at Figure S5.

### 2.7. Statistical Analysis

One-way ANOVA and post hoc statistical analysis using Bonferroni’s pairwise comparisons were used to determine statistical significance (*p < 0.05*).

### 2.8. Other methods

Retinal flat mounts were prepared as described previously [11,21].

## 3. Results

### 3.1. Effect of Loss of Vps34 in Rods on Cone Structure and Function

**Figure 1:**
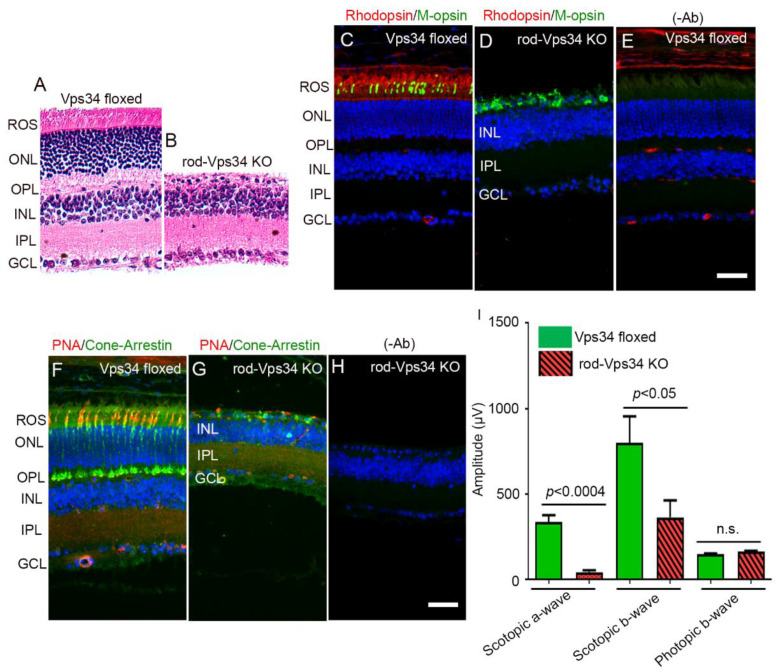
Effect of loss of Vps34 in rods on rod and cone structure. Rod photoreceptor-specific vacuole protein sorting 34 (Vps34) knockout mice were generated by breeding mice with a floxed Vps34 allele with mice that express Cre recombinase under the control of rhodopsin promoter. Morphologic examination of 8-week-old Vps34-floxed (**A**) and rod-Cre Vps34 KO (**B**) mice. ROS, rod outer segments; ONL, outer nuclear layer; OPL, outer plexiform layer; INL, inner nuclear layer; IPL, inner plexiform layer; GCL, ganglion cell layer. Scale bar = 50 μm. Prefer-fixed sections of 8-week-old Vps34-floxed (**C**,**E**,**F**) and rod-Vps34 KO (**D**,**G**,**H**) mouse retinas were subjected to immunofluorescence with rhodopsin (**C**,**D**), M-opsin (**C**,**D**), and cone-arrestin (**F**,**G**) antibodies. Panels (**F**,**G**) represent the immunostaining of sections with peanut agglutinin (PNA) lectin. Panels (**E**,**H**) represent the omission of the primary antibody. Scale bar = 50 μm. Scotopic a-wave, scotopic b-wave, and photopic b-wave electroretinographic analysis of retinas from 8-week-old Vps34-floxed and rod-Vps34 KO mice (**I**). Scotopic a-and b-wave amplitudes were measured at a flash intensity of 2.6 log cds/m^2^. Photopic b-wave amplitude was measured at a flash intensity of 3.3 log cd s/m^2^. Data are mean ± standard error of mean (SEM), *n* = 6. Significance, if any, is indicated on each panel.

In the majority of retinal degenerative diseases, loss of rod photoreceptors has a secondary effect on cones and promotes cone degeneration. The deletion of Vps34 in rods driven by a rhodopsin promoter resulted in massive rod degeneration by 8 weeks [12]. Figure 1A,B shows a complete loss of the photoreceptor layer in rod-Vps34 KO mice compared with wild-type mice (Vps34-floxed). Prefer-fixed retinal sections from Vps34-floxed and rod-Vps34 KO mice were stained for rhodopsin, cone opsin (M-opsin), cone arrestin, and PNA, the latter being used to label cone outer segments. The results showed that by 8 weeks, rhodopsin expression was absent from rod-Vps34 KO mice compared with Vps34-floxed mice (Figure 1C–E), whereas cone markers, M-opsin, cone arrestin, and PNA could still be observed in rod-Vps34 KO mice at 8 weeks in the rod-degenerated retina (Figure 1C–H). It is interesting to note that the cone structure, mainly the polarity, was lost in rod Vps34 KO mice due to the loss of photoreceptor layers, and the cones were present as a layer above the inner nuclear layer (Figure 1D,G). To determine whether the cones in rod-Vps34 KO mice were functional at 8 weeks, we performed electroretinography. There was no significant difference in photopic b-wave amplitudes between wild-type and rod-Vps34 KO mice (Figure 1I). However, there was a significant loss of scotopic a- and b-wave amplitudes in rod-Vps34 KO mice compared with Vps34-floxed mice (Figure 1I). These observations suggest that the loss of Vps34 in rods does not affect cone structure and function at 8 weeks.

### 3.2. Expression of Vps34 in the Cone-Dominant Retina

**Figure 2:**
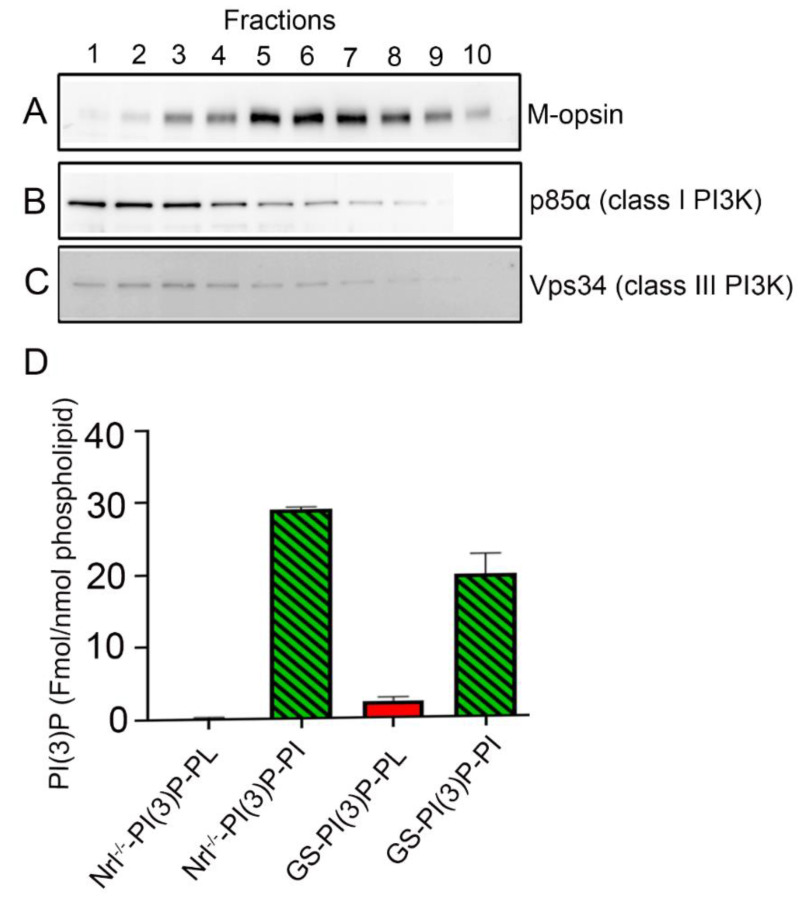
Expression of Vps34 in the cone-dominant retina. Fractions obtained by gradient centrifugation were probed with antibodies to M-opsin (**A**), the p85α subunit of class I phosphoinositide 3-kinase (PI3K) (**B**), and Vps34 (class III PI3K) (**C**). PI(3)P levels were measured from *Nrl^−/−^* and ground squirrel retina (**D**). Data are mean ± SEM (*n* = 3, *Nrl^−/−^*; *n* = 6, ground squirrel retina). Full-length blots are presented in the Supplementary Materials.

Rodent retinas are composed of more than 95% rods and less than 5% cones, making it difficult to study any protein in cones when the same protein is expressed in rods. Neural retina-specific leucine zipper protein (*Nrl*) is a transcriptional factor required for rod differentiation, and the absence of this protein leads to the development of cone-like photoreceptors [22]. The cone-like photoreceptors in *Nrl^−/−^* mouse retina are indistinguishable from wild-type mouse cones on the basis of several measures [23,24,25]. We took advantage of the *Nrl^−/−^* mouse retina, a mouse model of the cone-dominant retina, to study the expression of Vps34. Retinas were pooled from *Nrl^−/−^* mice, and low-speed supernatant was subjected to an OptiPrep density gradient to isolate cone outer segments with large portions of the cone inner segments attached. Collected fractions were examined for cone outer segment marker M-opsin (Figure 2A). Previous studies have shown that cells break open during sample preparation and release of soluble proteins. This occurs primarily in the inner segments, with the outer segments retaining their soluble components [12,17]. We examined these fractions for the presence of p85α subunit of class I PI3K (Figure 2B) and Vps34 (Figure 2C). Both class I and class III PI3K were found to be present in cone photoreceptors, and the fractionation suggested inner segment localization (Figure 2B,C).

### 3.3. PI(3)P Levels in the Cone-Dominant Retina

Our lipid extraction analysis clearly showed that almost all PI(3)P was extracted into an acid-soluble PI fraction. We found very little PI(3)P in the PL fraction of both *Nrl^−/−^* and ground squirrel retina. Our data showed that PI(3)P levels were higher in *Nrl^−/−^* retina than in ground squirrel [26] (Figure 2D). The results suggest that PI(3)P levels are higher in the cone-dominant retina.

### 3.4. The Functional Role of Vps34 in Cones

**Figure 3:**
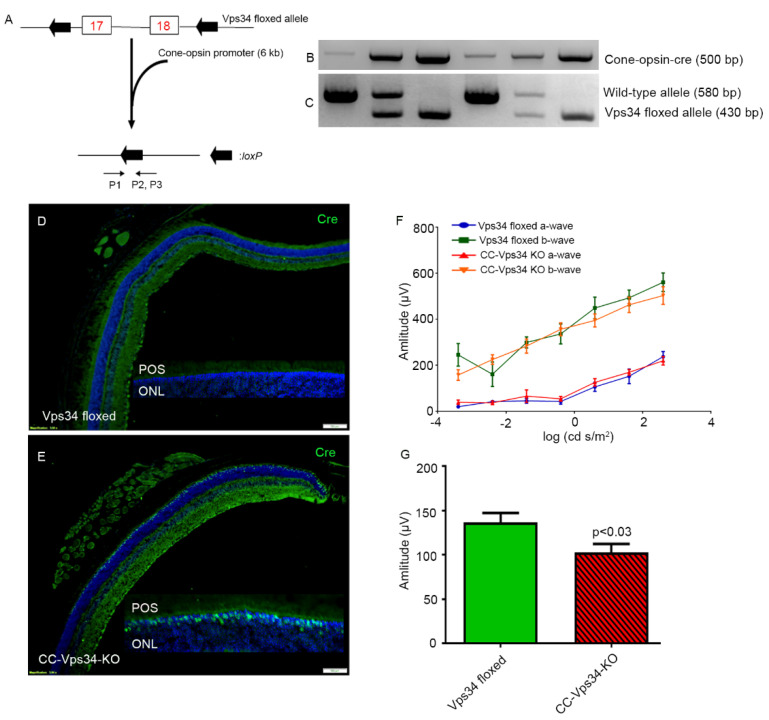
Characterization and retinal function of cone-conditional Vps34 KO mice. Schematic diagram of exon 17 and 18 floxed Vps34 loci (**A**). Cone photoreceptor-specific Vps34 knockout mice were generated by breeding mice with a floxed Vps34 with mice that express Cre recombinase under the control of human red/green opsin promoter (6.0 kb). Primer pairs P1, P2, and P3 were used to identify the wild-type and floxed Vps34 alleles (**A**). PCR diagnostic for cone opsin Cre (**B**) and floxed Vps34 and wild-type (WT) genes (**C**) was performed using mouse tail DNA samples. Immunohistochemical analysis of Cre recombinase immunolabeling was performed in Vps34-floxed (**D**) CC-Vps34 KO (**E**) retinas harvested from littermates. Inset: enlarged view between photoreceptor outer segments (POS) and outer nuclear layer (ONL). Scotopic a-wave (**F**), scotopic b-wave (**F**), and photopic b-wave (**G**) analyses were performed on 6-week-old Vps34-floxed and CC-Vps34 KO mice. Scotopic a-wave and scotopic b-wave amplitudes were carried out at different flash intensities (−3.4, −2.4, −1.4, −0.4, 0.6, 1.6, and 2.6 log cd s/m^2^). Photopic b-wave amplitudes were performed at a flash intensity of 3.3 log cd s/m^2^. Data are mean ± SEM (*n* = 16). Significance, if any, is indicated on each panel. Full-length blots are presented in the Supplementary Materials.

Cone photoreceptor-specific Vps34 knockout mice were generated by breeding mice with a floxed Vps34 with mice that express Cre recombinase under the control of human red/green opsin promoter (Figure 3A). The genotype of the CC-Vps34 KO mice was confirmed by PCR (Figure 3B,C). Since mice have rod-dominant retinas, in which rods outnumber cones, it is challenging to demonstrate a reduction in protein levels of Vps34 from total retinal extracts in cases where Vps34 is conditionally lost only in cones. To ensure the proper deletion of Vps34 in cones by our cone-expressing Cre line, we assessed Cre protein localization in the retinas of CC-Vps34 KO and Vps34-floxed littermates with immunofluorescence microscopy using an anti-Cre antibody. Cre expression was localized to cone photoreceptor nuclei in CC-Vps34 KO retinas (Figure 3E), but was absent from Vps34-floxed mice (Figure 3D).

Electroretinography (ERG) was carried out when the mice were six weeks old. We found no significant difference in scotopic a-wave and scotopic b-wave amplitudes between Vps34-floxed and CC-Vps34 KO mice (Figure 3F). However, the photopic b-wave amplitude in CC-Vps34 KO mice was significantly reduced compared with that in Vps34-floxed mice (Figure 3G). These observations suggest that Vps34 is required for cone function.

**Figure 4:**
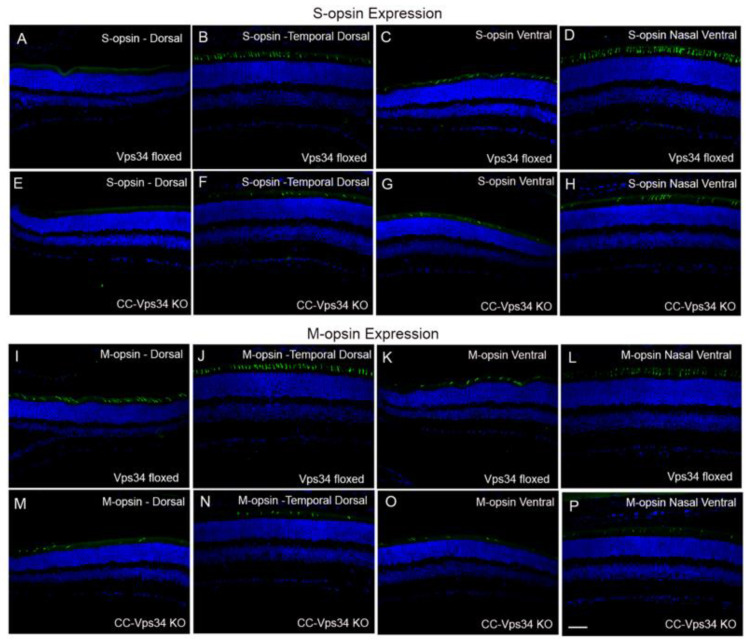
Regional distribution of S-opsin- and M-opsin-positive cones in Vps34 floxed and CC-Vps34 KO mice. The difference in expression of S-opsin (S-opsin-positive cones) (**A**–**H**) and M-opsin (M-opsin-positive cones) (**I**–**P**) in dorsal, temporal dorsal, ventral, and nasal ventral areas of the retina from 6-week-old Vps34-floxed and CC-Vps34 KO mice. The images shown are representative of six retinas examined from Vps34-floxed and CC-Vps34 KO mice. Scale bar = 50 μm.

**Figure 5:**
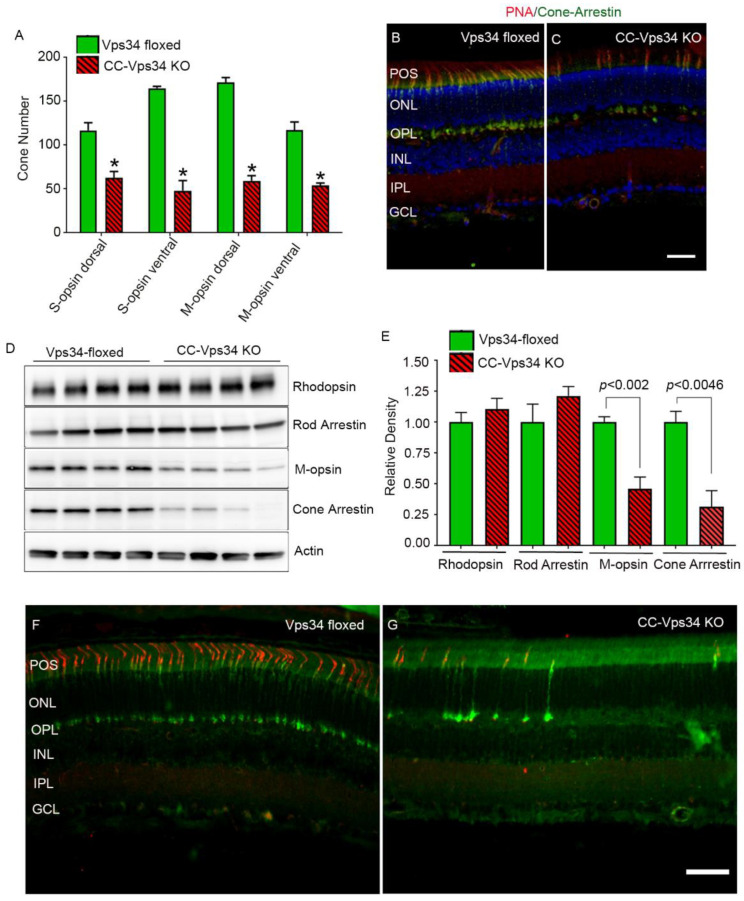
Structural characterization of Vps34-floxed and CC-Vps34 KO mouse retina. Quantification of the number of S-opsin and M-opsin positive cones in dorsal and ventral regions of the retina counted starting from the optic nerve head (**A**). Data are mean ± SEM (*n* = 6). Significance, if any, is indicated on each panel. Prefer-fixed sections of 6-week-old Vps34-floxed and CC-Vps34 KO mouse retinas were subjected to immunofluorescence with PNA lectin (red) and anti-cone arrestin (green) antibody. * *p* < 0.05 (**B**,**C**). Scale bar = 50 μm. Retinal homogenates (5.0 μg protein) from four independent 6-week-old Vps34-floxed and CC-Vps34 KO mice were subjected to immunoblot analysis with anti-rhodopsin, anti-rod arrestin, anti-M-opsin, anti-cone arrestin, and anti-actin antibodies (**D**). The photoreceptor protein expression was normalized to actin (**E**). Data are mean ± SEM (*n* = 4). Significance, if any, is indicated on each panel. Prefer-fixed sections of 32-week-old Vps34-floxed and CC-Vps34 KO mouse retinas were subjected to immunofluorescence with PNA lectin (red) and anti-cone arrestin (green) antibody (**F**,**G**). POS, photoreceptor outer segments; ONL, outer nuclear layer; OPL, outer plexiform layer; INL, inner nuclear layer; IPL, inner plexiform layer; GCL, ganglion cells. Scale bar = 50 μm. Full-length blots are presented in the Supplementary Materials.

The reduced cone function in CC-Vps34 KO mice could be due to a functional deficit that does not affect the structure of cones or to a decreased number of cones because of cone degeneration. To examine this possibility, we determined the expression of both short-wavelength (S-opsin) and medium-wavelength opsins (M-opsin) in Vps34-floxed and CC-Vps34 KO mouse retinas (Figures S1 and S2). S-opsin-positive cones are predominant in the ventral region of the retina compared with the dorsal region, whereas M-opsin-positive cones are distributed in both dorsal and ventral regions, but have slightly higher distribution in the dorsal region [27]. We present the data as dorsal, temporal dorsal, ventral, and nasal ventral, which show a loss of both S- and M-opsin-positive cones in all of these regions of CC-Vps34 KO mouse retinas compared with Vps34-floxed mouse retinas (Figure 4). The number of S- and M-opsin-positive cones were counted across the entire retina and in specific regions (dorsal and ventral) through the optic nerve. The results indicate that both S-opsin- and M-opsin-positive cones were significantly reduced in CC-Vps34 KO mouse retinas compared with Vps34-floxed mouse retinas (Figure 5A). The degeneration started around 1 month and continued to degenerate until 8 months. We found the loss of M-opsin-positive cones preceded the loss of S-opsin-positive cones.

To determine whether loss of Vps34 in cones resulted in cone degeneration, we stained the retinal sections with PNA, which labels cone outer segment sheets, and with cone arrestin, which labels cone inner segments. The results indicated that there were fewer PNA- and cone arrestin-labeled cones in CC-Vps34 KO mice than in Vps34-floxed mice (Figure 5B,C). To corroborate our immunohistochemistry results, we carried out immunoblot analysis on Vps34-floxed and CC-Vps34 KO mouse retinal lysates with rhodopsin, rod arrestin, M-opsin, cone arrestin, and actin antibodies (Figure 5D), and normalized the protein levels to actin (Figure 5E). The results showed significantly decreased levels of cone markers M-opsin and cone arrestin in CC-Vps34 KO mouse retinas compared with Vps34-floxed mouse retinas (Figure 5E). There were no significant differences in the levels of rhodopsin or rod arrestin between CC-Vps34 KO and Vps34-floxed retinas (Figure 5D,E). Sections from 32-week-old mouse retinas were stained with PNA and cone arrestin and the results show that there were fewer PNA- and cone arrestin-labeled cones in CC-Vps34 KO mice than in Vps34-floxed mice (Figure 5F,G). Collectively, these findings suggest that a loss of Vps34 in cones results in cone degeneration.

**Figure 6:**
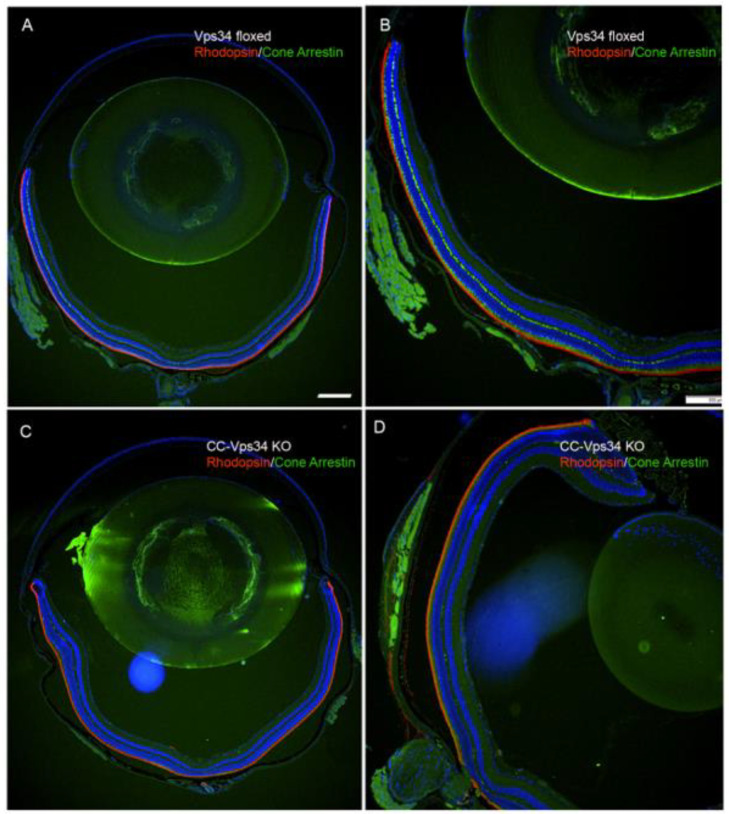
The loss of Vps34 in cones did not affect rod structure. Prefer-fixed sections of 6-week-old Vps34-floxed and CC-Vps34 KO mouse retinas were subjected to immunofluorescence with anti-rhodopsin (red) and anti-cone arrestin (green) antibodies. Images were captured from dorsal and ventral regions of the retina (**A**,**C**). The dorsal region of the retina was captured at higher magnification (**B**,**D**). Scale bar = 100 μm.

To determine whether the loss of Vps34 in cones has any effect on rods, we stained the retinal sections with rhodopsin and cone arrestin. Our immunohistochemistry results showed no difference in the expression of rhodopsin in the entire retina between Vps34-floxed and CC-Vps34 KO mouse retinas (Figure 6A–D). However, cone arrestin-positive cones were absent from CC-Vps34 KO mouse retinas (Figure 6C,D), whereas cone arrestin-positive cones were well preserved in Vps34-floxed mouse retinas (Figure 6A,B). The overall morphology of the retina was indistinguishable between Vps34-floxed and CC-Vps 34 KO mice at 1, 5, and 8 months of age (Figure S3). We previously observed that the cone-specific deletion of a glycolytic enzyme pyruvate kinase M2 isoform results in changes in the gene expression in Müller cells [28]. In this study, in order to determine whether loss of Vps34 in cones has any effect on Müller cells, we stained Vps34-floxed and CC-Vps34 KO mouse retinal sections with Müller cell markers, GS, and GFAP. We found no change in the expression of these two markers between Vps34-floxed and CC-Vps34 KO mouse retinas (Figure S4).

## 4. Discussion

Phosphoinositides with phosphates at the 3-positions can be generated from the action of all three classes of PI3Ks [29]. Vps34-generated lipid is mainly involved in the recruitment of proteins containing PI(3)P-binding domains to intracellular membranes, where PI(3)P is involved in the initiation and maturation of autophagosomes [30]. Vps34 has also been involved in other signaling processes, such as nutrient sensing in the mammalian target of rapamycin pathway in mammalian cells [31,32].

Published literature on Vps34 suggests that it is essential for cardiac functions, as ablation has been shown to result in reduced contractility of the heart muscle and cardiomegaly [33]. Mice lacking Vps34 in the liver exhibit impaired protein turnover, hepatomegaly, and hepatic steatosis [33]. The deletion of Vps34 in muscle has been shown to result in muscular dystrophy [34], and its ablation in sensory neurons has been shown to result in rapid neurodegeneration due to a defect in the endosomal pathway, without affecting the autophagic pathway [14]. Vps34 is necessary for the proper function of the proximal kidney tubules, observed as a blockage in autophagic flux and impaired apical trafficking, resulting in renal insufficiency [35].

Rod photoreceptor cells lacking Vps34 exhibit a defect in the fusion of endosomal and autophagosomal membranes with lysosomes and abnormal accumulation of membrane structures in rods [12]. Interestingly, at an early age, Vps34 KO rods have been shown to display normal structure and function and rhodopsin trafficking, but by 12 weeks, the rods undergo a progressive retinal degeneration [12]. In the current study, we observed that loss of Vps34 in cones results in the loss of structure and function that lead to cone degeneration.

We previously generated cone-conditional knockouts of the two subunits of class I PI3K (p85α and p110α), which phosphorylates PI(4,5)P_2_ to PI(3,4,5)P_3_. Both exhibited an age-related cone degeneration starting at around 3 months of age [10,11]. In the present study, CC-Vps34 KO mice showed an earlier onset of cone degeneration, and a majority of cones had degenerated by 1.5 months. Although temporally different, these two degenerations may share some common biochemical features. The Vps34-generated PI(3)P interacts with FYVE (named for the first four proteins in which it was recognized, Fab1p, YOTB, Vac1p, and EEA1) domain-containing proteins [36]. One of the FYVE domain-containing proteins is phosphatidylinositol 3-phosphate 5-kinase (PIKfyve) [36]. PIKfyve tethers to PI(3)P at the membrane and phosphorylates PI to PI(5)P and PI(3)P to PI(3,5)P_2_ [36]. PI(5)P is phosphorylated by the enzyme type II phosphatidylinositol 5-phosphate 4-kinase (PIP4K) to generate PI(4,5)P_2_ [5], and this substrate is phosphorylated by class I PI3K to generate PI(3,4,5)P_3_ [1,6]. We speculate that PI(3)P-generated PI(5)P might play an important role in cone photoreceptors. Both PI(4,5)P_2_ and PI(3,4,5)P_3_ are important molecules needed for cellular functions.

Age-related macular degeneration (AMD) and diabetic retinopathy are the most common retinal diseases affecting cones that result in cone degeneration [37,38,39,40,41]. In cone and cone-rod dystrophies, cone degeneration occurs progressively [42]. PI(3)P plays an important role in the regulation of autophagy [43], and autophagy has been shown to support color vision [44]. The structural and functional phenotypes observed in mouse cones lacking Vps34/PI(3)P could be attributable to defects in autophagy. Further studies are needed to understand the molecular mechanism of cone cell death in cone-conditional Vps34 knockout mice. Reactive gliosis has been observed in various retinal diseases including AMD, diabetic retinopathy, glaucoma, retinal detachment, and retinitis pigmentosa [45]. Surprisingly, we did not observe any Müller cell activation in cone-conditional Vps34 KO mice. Interestingly, we found PI(3)P lipid in primary Müller cells isolated from mouse retina (Rajala, unpublished data). It may be possible that Müller cell PI(3)P could be compensating for the loss of PI(3)P in cones. Further studies are needed to determine the role of PI(3)P in retinal gliosis.

The mechanism of cone degeneration in cone-conditional Vps34 KO mice is yet to be studied. Cone photoreceptors constitute a small percentage of total retinal photoreceptors [46,47], and it is technically challenging to study the cone-specific expression of a protein that is also expressed in the rods. Due to this technical difficulty, we were not able to quantify the loss of Vps34 protein and PI(3)P levels in cones. We examined the expression of Cre recombinase as an indirect measure of Vps34 deletion in cones. We are in the process of generating Nrl/CC- Vps34 double KO mice and cone-conditional PIKfyve KO mice to study the mechanism of cone degeneration in cone conditional Vps34 KO mice.

## 5. Conclusions

The major pathway for the generation of the low-abundance membrane lipid PI(3)P synthesis is catalyzed by class III phosphoinositide 3-kinase (PI3K) Vps34. Both PI(3)P and Vps34 protein are present in mouse cones. The deletion of Vps34 in mouse cones resulted in the loss of cone structure and function, resulting in an early onset of cone degeneration. Our studies indicate that class III PI3K, and presumably PI(3)P, play essential roles in cone photoreceptor cell function and survival.

## Supplementary Materials

The following are available online at https://www.mdpi.com/2079-7737/9/11/384/s1, Figure S1: Expression of S-opsin in Vps34-floxed and CC-Vps34 KO mice. Figure S2: Expression of M-opsin in Vps34-floxed and CC-Vps34 KO mice. Figure S3: Morphology of cone-specific Vps34 KO retina and assessment of rod outer segment integrity. Figure S4: Expression of glial fibrillary acidic protein (GFAP) and glutamine synthetase (GS) in cone-conditional Vps34 KO mice. Figure S5: Original Blots.
