# Supplementary material for: Loss of Class III Phosphoinositide 3-Kinase Vps34 Results in Cone Degeneration
Source: Biology (Basel). 2020 Nov 7;9(11):384. doi: 10.3390/biology9110384 (PMC7695136; doi:10.3390/biology9110384)
Supplement: Supplementary file 1 [file biology-09-00384-s001.pdf]

Supplementary Materials:

Loss of Class III Phosphoinositide 3-Kinase Vps34 Results in Cone Degeneration

Ammaji Rajala, Feng He, Robert E. Anderson, Theodore G. Wensel and Raju V. S. Rajala

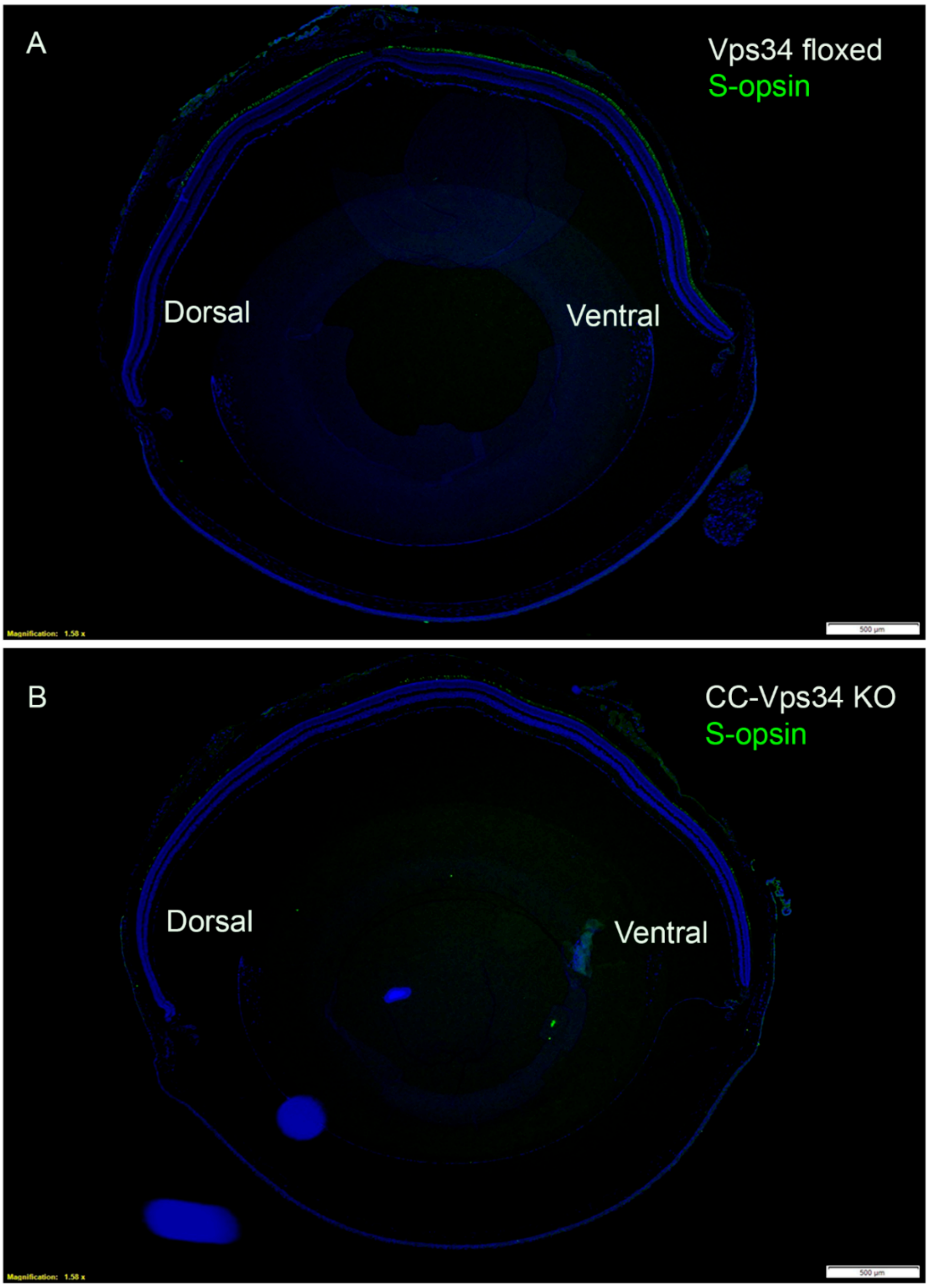

Figure S1. Expression of S-opsin in Vps34 floxed and CC-Vps34 KO mice. Prefer-fixed sections of 6-week-old Vps34 floxed (**A**) and CC-Vps34 KO (**B**) mouse retinas were subjected to immunofluorescence with S-opsin antibody.

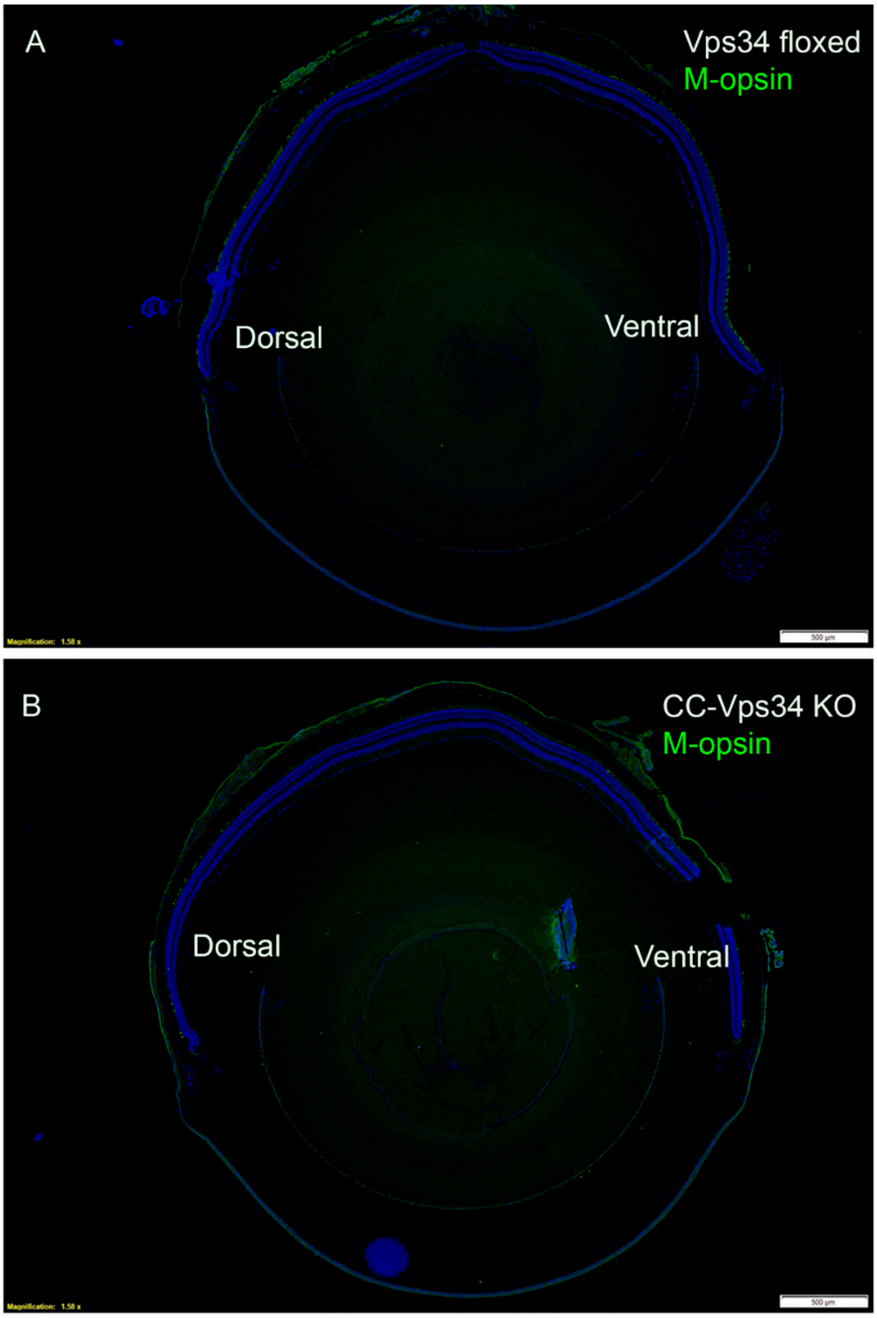

Figure S2. Expression of M-opsin in Vps34 floxed and CC-Vps34 KO mice. Prefer-fixed sections of 6-week-old Vps34 floxed (**A**) and CC-Vps34 KO (**B**) mouse retinas were subjected to immunofluorescence with M-opsin antibody.

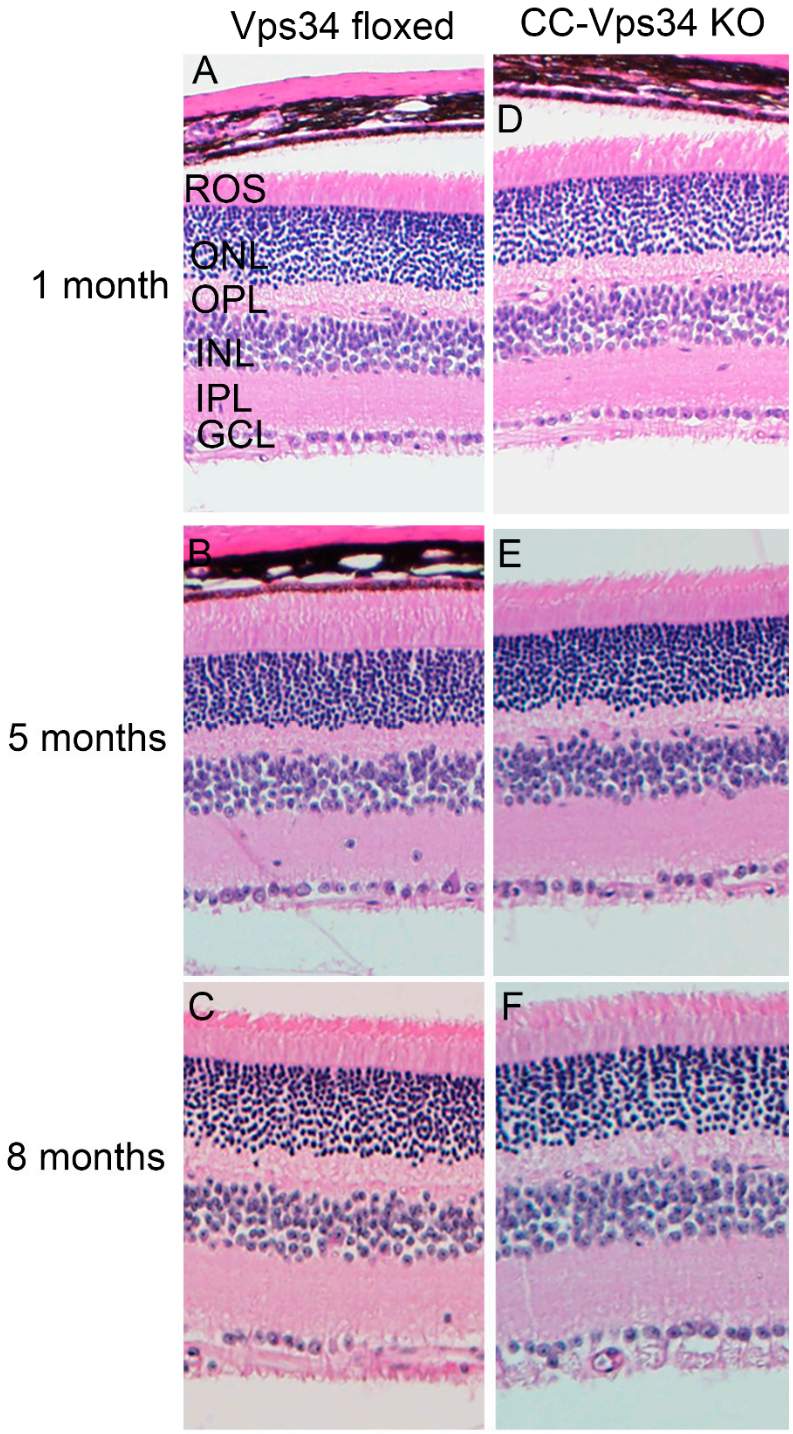

Figure S3. Morphology of cone-specific Vps34 KO retina and assessment of rod outer segment integrity. Morphologic examination of retinas from Vps34 floxed (**A-C**) and CC-Vps34 KO (**D-F**) mice at 1 (**A, D**), 5 (**B, E**), and 8 (**C, F**) months of age. ROS, rod outer segments, ONL, outer nuclear layer, OPL, outer plexiform layer, INL, inner nuclear layer, IPL, inner plexiform layer, GCL, ganglion cell layer.

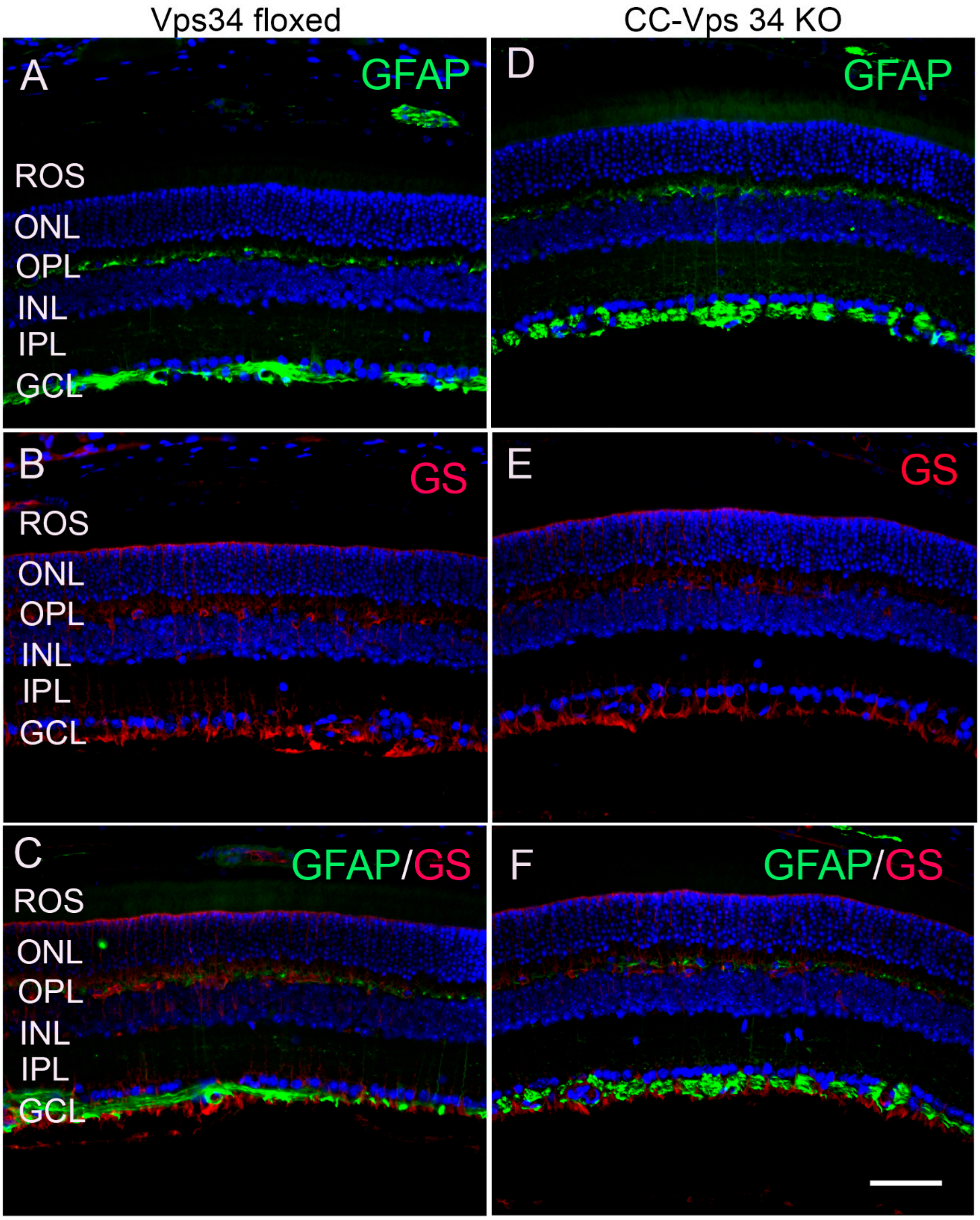

Figure S4. Expression of GFAP and GS in cone-conditional Vps34 KO mice. Prefer-fixed sections of 6-week-old Vps34 floxed (**A–C**) and CC-Vps34 KO (**D–F**) mouse retinas were subjected to immunofluorescence with GFAP (green, **A, D**) and GS (red, **B, E**) antibodies. Panel **C** and **F** represent the merge images of GFAP and GS. ROS, rod outer segments, ONL, outer nuclear layer, OPL, outer plexiform layer, INL, inner nuclear layer, IPL, inner plexiform layer, GCL, ganglion cell layer. Scale bar = 50 μm.

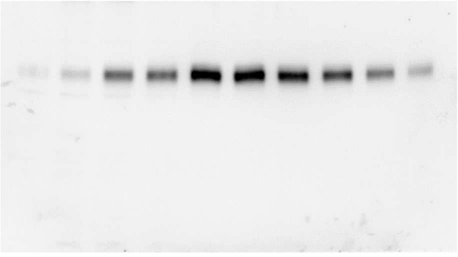

Full-length blot of Figure 2 (A) in the main text (M-opsin)

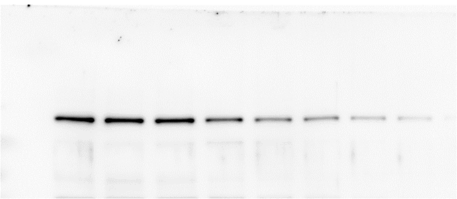

Full-length blot of Figure 2 (B) in the main text (p85α (class I PI3K)

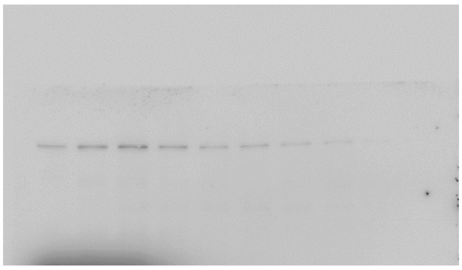

Full-length blot of Figure 2 (C) in the main text (Vps34 (class III PI3K)

Original blots for Figure 2

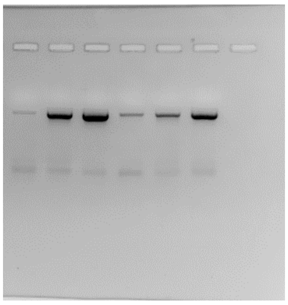

Full-length blot of Figure 3 (B) in the main text (Cone-opsin-cre)

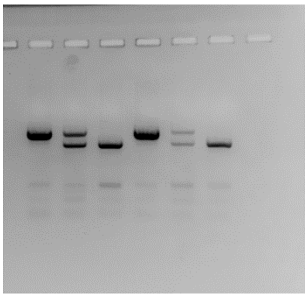

Full-length blot of Figure 3 (C) in the main text (Wild-type and Vps34 floxed allele)

Original blots for Figure 3

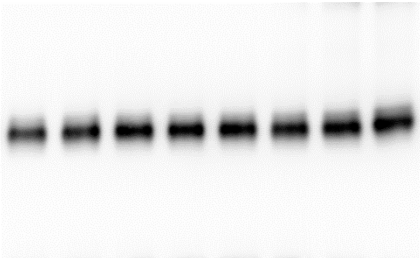

Full-length blot of Figure 5 (D) in the main text (Rhodopsin)

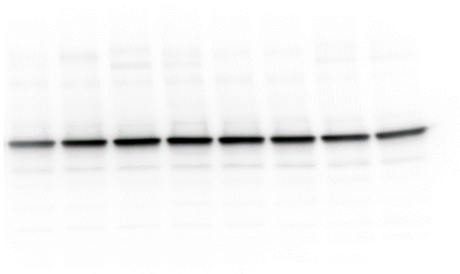

Full-length blot of Figure 5 (D) in the main text (Rod Arrestin)

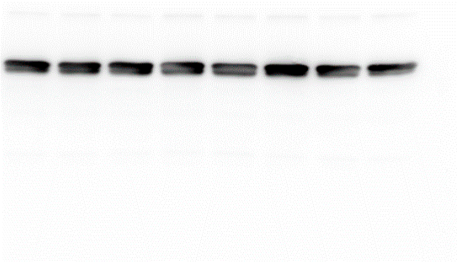

Full-length blot of Figure 4 (5) in the main text (Actin)

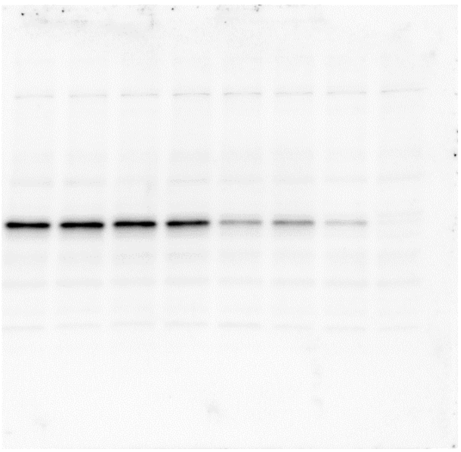

Full-length blot of Figure 5 (D) in the main text (Cone Arrestin)

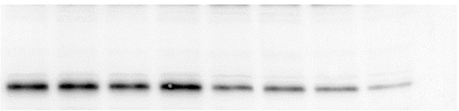

Full-length blot of Figure 5 (D) in the main text (M-opsin)

Original blots for Figure 5

Figure S5. Original blots.

© 2020 by the authors. Licensee MDPI, Basel, Switzerland. This article is an open access article distributed under the terms and conditions of the Creative Commons Attribution (CC BY) license (http://creativecommons.org/licenses/by/4.0/).
